# 

**Published:** 1811-03

**Authors:** 


### MONTHLY CATALOGUE OF MEDICAL BOOKS.

Observations on the best Mode of relieving Strictures in the Urethra, with Remarks on the frequent Inefficacy and ill effects of Caustic Applications. By William Wadd. 8vo. boards. Callow.

---

Practical Observations on the Formation of an Artificial Pupil in several deranged States of the Eye; to which are annexed, Remarks on the Extraction of soft Cataracts and those of the membranous Kind, through a Puncture in the Cornea. Illustrated by Plates. By Benjamin Gibson. 8vo. boards. Cadell and Davies.

A Con-

A Conspectus of the London, Edinburgh, and Dublin Pharmacopœias, in which are clearly explained the Virtues of each Article and Medicine, and the Doses and Diseases for which the several Remedies therein contained are employed. By E. G. Clarke, M. D. 18mo. sewed. Cox.

---

The Modern Surgeon, or plain and rational Rules for the Direction of Practice, founded on the Observations and Experience of the most distinguished Practitioners. 8vo. boards. Murray.

---

Natural History of the Human Teeth, with a Treatise on their Diseases from Infancy to Old Age, adapted for general Information. To which are added, Observations on the Physiognomy of the Teeth, and of the Projecting Chin, with Two Engravings. By Joseph Murphy, 8vo. boards. Callow.

---

Very lately there was a fall of meteoric stones in Caswell county, New Connecticut. Their descent was seen for a considerable distance round, and two reports distinctly heard at Hillsborough, a distance of thirty miles. A fragment weighing a pound and three quarters, struck a tree in the new ground of a Mr. Taylor, near to which some woodcutters were at work, who ran home in alarm. On recovering from their fright they returned to the place, and brought away the stone which was still hot. It is of a dark brown colour, porous, and contains iron.

It is stated very positively, in a recent publication, that horse-flesh now forms a common article for food in Norway: and that since the year 1803, there have been slaughtered at Christiana 400 horses for the consumption of that town.

At the last fair at Leipsic, the number of German books brought for sale, amounted to between 1,000 and 1,100. These were for the most part, either compilations, or insignificant works.

### NOTICES TO CORRESPONDENTS.

Dr. Harrison of Thornbury, has deemed it necessary, in consequence of the incredulity of '*Senex*,' to send us the following statement, sworn before the Rev. R. Slade, Vicar of Thornbury, and Justice of the Peace for Gloucestershire.

" To remove every suspicion of imposition of relating a falsehood with any intention to impose on any man or any set of men whatsoever, I declare upon oath on the Evangelists of Almighty God that the case related to me by my son T. E. Harrison, late surgeon of his Majesty's ship Halifax, in your useful Medical Journal for January, 1811, (P. 31.) on the fatal effects of Cherries, I believe to be true, and can safely declare that I know it to be so.'

We have copied this affidavit, because Dr. Harrison seems hurt that his former account should have been disputed ; but we cannot sanction appeals to magistrates on such occasions ; scarcely a nostrum is advertised without a case sworn before a justice or a clergyman ; and this distinction between regulars and quacks is in our opinion a wholesome one.

Dr. Harrison has also related some fatal instances of copious bleeding in fever ; and one of boiling water being poured on the breast, and afterwards " live wood embers out of the fire" being laid upon it, to raise a blister. As they are not stated with sufficient minuteness, we suppose the Doctor did not intend them for insertion.

We have been favoured with communications from I. F. R. from *Senex*, from Messrs. Eagland and Cullen ; and from Dr. Ireland.

*Printed by E. Hemsted, Great New Street, Fetter Lane.*

